# Low-Cycle Fatigue Damage Mechanism and Life Prediction of High-Strength Compacted Graphite Cast Iron at Different Temperatures

**DOI:** 10.3390/ma17174266

**Published:** 2024-08-28

**Authors:** Qihua Wu, Bingzhi Tan, Jianchao Pang, Feng Shi, Ailong Jiang, Chenglu Zou, Yunji Zhang, Shouxin Li, Yanyan Zhang, Xiaowu Li, Zhefeng Zhang

**Affiliations:** 1 State Key Laboratory of Engine and Powertrain System, Weichai Power Co., Ltd., 197A Fushou East Street, Weifang 261061, China; wuqh@weichai.com (Q.W.); jiangal@weichai.com (A.J.); zhangyunji@weichai.com (Y.Z.); 2 Shenyang National Laboratory for Materials Science, Institute of Metal Research, Chinese Academy of Sciences, Shenyang 110016, China; tanbingzhi8899@163.com (B.T.); clzou@imr.ac.cn (C.Z.); shxli@imr.ac.cn (S.L.); yyzhang@imr.ac.cn (Y.Z.); 3 Department of Material Physics and Chemistry, School of Materials Science and Engineering, and Key Laboratory for Anisotropy and Texture of Materials (Ministry of Education), Northeastern University, Shenyang 110819, China; shifeng@imp.neu.edu.cn (F.S.); xwli@mail.neu.edu.cn (X.L.)

**Keywords:** compacted graphite cast iron, microstructures, experimental temperature, low-cycle fatigue, damage mechanism, life prediction

## Abstract

Tensile and low-cycle fatigue tests of high-strength compacted graphite cast iron (CGI, RuT450) were carried out at 25 °C, 400 °C, and 500 °C, respectively. The results show that with the increase in temperature, the tensile strength decreases slowly and then decreases rapidly. The fatigue life decreases, and the life reduction increases at high temperature and high strain amplitude. The oxide layer appears around the graphite and cracks at high temperature, and the dependence of crack propagation on ferrite gradually decreases. With the increase in strain amplitude, the initial cyclic stress of compacted graphite cast iron increases at three temperatures, and the cyclic hardening phenomenon is obvious. The fatigue life prediction method based on the energy method and damage mechanism for compacted graphite cast iron is summarized and proposed after comparing and analyzing a large amount of fatigue data.

## 1. Introduction

Internal combustion engines are gradually developing in the direction of higher power, lower energy consumption, reduced pollution, decreased noise, and improved durability. As the core area of energy conversion, the combustion chamber directly affects the efficiency of the engine [1,2,3,4]. The cylinder head in the combustion chamber needs to bear huge mechanical and thermal loads during operation [5,6,7,8,9,10]. The harsh working environment of alternating hot and cold working conditions and dynamic and static load conversion in the combustion chamber makes the failure of the cylinder head easier and even reduces its service life.

Compacted graphite cast iron (CGI) is one of the main materials used in cylinder heads. Compared with other cast iron materials, CGI has comprehensive mechanical and physical properties, such as good casting performance and thermal conductivity and high strength, and it is widely used for components in working environments of high mechanical and thermal stresses; one of the most typical components is the cylinder head of large diesel engines [11,12,13,14,15]. To identify the cause of the failure of the component, many studies on the mechanical properties of cast iron materials have been carried out; the effects of microstructure and temperature on tensile properties, high-cycle fatigue (HCF), and fatigue damage behavior have been studied [16,17,18,19,20]. In particular, attention has been paid to low-cycle fatigue (LCF), including softening/hardening phenomena, damage mechanisms, and life prediction models [21,22]. Qiu et al. found that CGI (RuT300) has the longest fatigue life and the smallest stable hysteresis loop area at 400 °C [23]. Pevec et al. found that the strain of gray cast iron shows slight softening at the end of its fatigue life, with cyclic softening becoming more obvious with increases in temperature [24]. The corresponding influence mechanism of temperature is mainly achieved by affecting the effective bearing size of ferrite. In addition, it can be seen that there are many studies on the mechanical properties of cast iron, but few on CGI with high strength. In addition to fatigue behavior, it is also very important to find a suitable LCF life prediction method for compacted graphite cast iron.

By considering the stress amplitude, strain amplitude, hysteresis energy, and other factors of the material, the fatigue life has been predicted. The Basquin model is mainly based on the relationship between stress amplitude and fatigue life, which is effectively used for HCF [25]. The Coffin–Manson model is based on the strain–life curve, which is mainly used for LCF [26]. Combining the effect of strain and stress, the energy method shows obvious advantages and has been continuously developed and widely applied to fatigue life prediction [27,28,29,30]. The LCF process as an energy (thermal energy, elastic–plastic energy) dissipation process and a cumulative damage process has been interpreted for the accurate prediction of fatigue life based on hysteresis energy applied to many materials [31,32]. However, the influence of stress amplitude or strain amplitude on fatigue life is mainly considered, and other factors such as temperature and the microstructure of materials are not considered, which makes the prediction results deviate greatly. Therefore, whether the existing fatigue life prediction model can be applied to component materials with complex microstructures and service environments needs to be re-evaluated. 

As mentioned above, to improve the combustion efficiency of internal combustion engines, higher-strength materials need to be used for safe service. As a high-strength CGI material, RuT450 was chosen; the LCF properties of CGI were measured and analyzed at different temperatures (25 °C, 400 °C, and 500 °C) and the corresponding damage mechanism and the evaluation model were discussed.

## 2. Experimental Materials and Procedures

### 2.1. Research Material

In this experiment, the as-cast CGI (RuT450) with a ferrite and pearlite matrix microstructure was directly cut from the combustion surface of a diesel engine cylinder head. Compacted graphite cast iron is produced by German thermal analysis control technology. In the actual production process, a 12 t coreless induction furnace (ABP Induction System Co., Ltd., Dortmund, Germany) was used to melt the iron. After the iron was melted, a thermal analysis test was carried out. According to the test results, the chemical composition was adjusted. When the chemical composition was adjusted to the appropriate value, a 2.5 t Heinrich Wagner Sinto (HWS) ladle was used for pouring, and wire feeding equipment was used for vermicularizing and inoculation. The important chemical composition was measured and is shown in Table 1.

### 2.2. Microstructure Analysis

**Figure 1:**
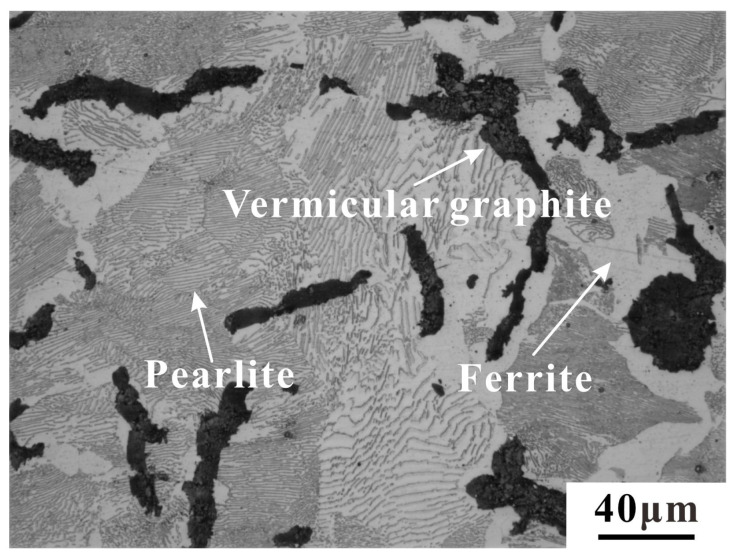
Microstructure of RuT450.

**Figure 2:**
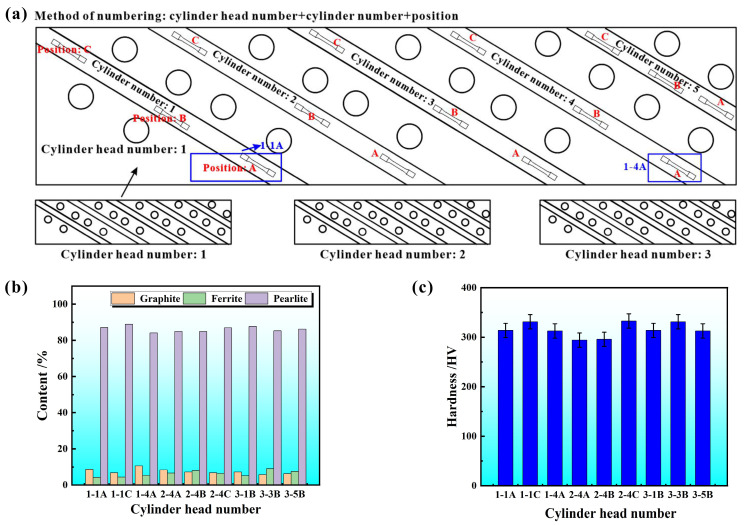
The schematic numbering method (**a**), microstructure (**b**) and hardness (**c**) statistics of different positions of different cylinder heads.

Microstructure uniformity is very important for analyzing the results. Thin slices with the size of 2 mm × 4 mm × 5 mm were cut to observe microstructure. To avoid the influence of scratches and the residual oxide layer during the cutting process, the surface of the original sample was polished using 400#, 800#, 1000#, 1200#, and 2000# sandpaper, and was further polished with W2.5 grinding paste and then corroded by a solution containing 4% nitric acid and 96% ethanol for 8 s. The microstructure of RuT450 was observed by OLYMPUS−DP73 (Olympus Corporation, Tokyo, Japan) laser confocal microscope. Image−Pro−Plus (IPP) 6.0 was utilized for quantitative analysis of the phase contents. The typical microstructure consists of graphite, ferrite, and pearlite, as shown in Figure 1. Nine groups of samples taken from different positions on the different cylinder heads were chosen for analysis. The specific naming rules are shown in Figure 2a, the first and second numbers representing cylinder head number and cylinder position one, the third letter representing the number of the sampling location. The uniformity of the phase contents of RuT450 is shown in Figure 2b. In accordance with the state standard of the People’s Republic of China [33], IPP was used to determine the area percentage of graphite, pearlite, and ferrite. The calculation method for vermicularity is detailed in Equation (1).

(1) $$\rho_{V}=\frac{\;\sum A_{V}+0.5\times A_{f}}{\sum A_{i}}\times 100$$

Here, $\rho_{V}$ is the vermicularity, *A*_V_ is the area of vermicular graphite particles, *A*_f_ is the area of flocculent graphite particles, and *A*_i_ is the area of each graphite particle.

According to the calculations (Equation (1)), the vermicularity of RuT450 is 84.5%, and the area percentage of graphite is 8.38%, including compacted and spherical forms. Ferrite is milky white under an optical microscope, the area ratio is about 6.62%. The pearlite is gray-brown, and the area ratio is approximately 85.00%.

### 2.3. Mechanical Properties

Hardness changes can conveniently reflect the differences in material microstructure and can be quickly assessed through a simple method. The micro-Vickers hardness test was conducted on the uncorroded samples, and the specific results are shown in Figure 2c. The error ranges between metallographic structure and Vickers hardness are within 5%, indicating a small difference in microstructure, which ensures uniformity.

**Figure 3:**
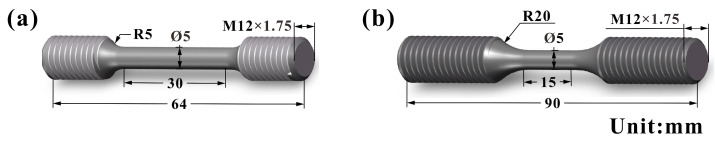
Shape and size of (**a**) tensile specimen and (**b**) fatigue specimen (unit mm).

According to the actual working conditions of the engine cylinder head, the tensile and fatigue experimental temperatures were selected as 25, 400, and 500 °C. During the tensile and LCF experiments at 400 and 500 °C, the samples were first heated to a preset temperature by a resistance furnace and were kept for 30 min, and then the corresponding experiments were carried out. The axial loading tensile test of RuT450 was carried out on the Instron 8862 universal testing machine (Instron Corporation, Boston, MA, USA). The constant engineering strain rate is 5 × 10^−4^s^−1^, and at least three samples were tested at each temperature. The sizes of the tensile and fatigue samples are shown in Figure 3. The LCF test with symmetric axial loading was performed using the Instron 8801 hydraulic servo fatigue testing machine (Instron Corporation, Boston, MA, USA). The control mode of total strain amplitude is used. The strain is measured by the extensometer. The strain loading waveform is triangular, and the strain rate is 1 × 10^−2^s^−1^. The loaded strain amplitudes are ±0.1%, ±0.15%, ±0.2%, and ±0.25% at different temperatures. If the specimen fails during the loading process, the experiment stops; if the sample does not break when the cycle reaches 10^5^ during loading, the experiment stops, and the sample is marked as suspension. After the LCF test, the fatigue fractures were cleaned ultrasonically with alcohol, and then the front and longitudinal sections of the fatigue fractures were observed by Hitachi JSM−6510 scanning electron microscope (Japan Electronics Co., Ltd., Tokyo, Japan).

## 3. Results

### 3.1. Tensile Properties

**Figure 4:**
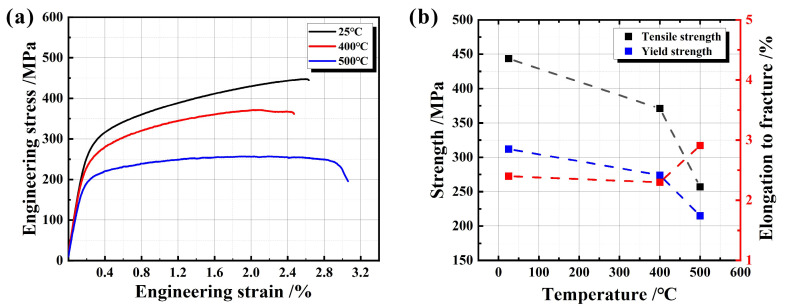
(**a**) The tensile stress−strain curves and (**b**) the changing trend of tensile strength, yield strength, and elongation to fracture of RuT450.

At 25 °C, 400 °C, and 500 °C, the engineering stress−strain curves, strength data, and elongation to fracture of RuT450 are depicted in Figure 4. Figure 4a shows that there is no obvious yield point at the three different temperatures, consistent with the description in the references [34]. At high temperatures, the curve exhibits slight fluctuations, reflecting the plastic flow phenomenon during the tensile process. Further analysis in Figure 4b reveals that the elongation to fracture increases slightly(an increase of 0.51%), while the tensile strength and yield strength decrease with the increase in experimental temperature. The downward trend becomes more pronounced when the temperature exceeds 400 °C. Therefore, the specific temperature point of 400 °C is defined as the critical transition temperature Tc. When the test temperature is higher than the critical transition temperature Tc, the reduction rate of tensile strength is significantly faster than that of yield strength. At 500 °C, the yield strength approaches the tensile strength. This phenomenon indicates that the yield ratio (the ratio of yield strength to tensile strength) remains relatively stable before the critical transition temperature (yield ratio at 25 °C: 0.7, 400 °C: 0.73). Once the temperature surpasses this point, the yield ratio increases rapidly (yield ratio at 500 °C: 0.84). It is evident that both tensile strength and yield strength are temperature-sensitive, and this sensitivity becomes more pronounced after surpassing the critical transition temperature.

### 3.2. Tensile Fracture Morphologies

**Figure 5:**
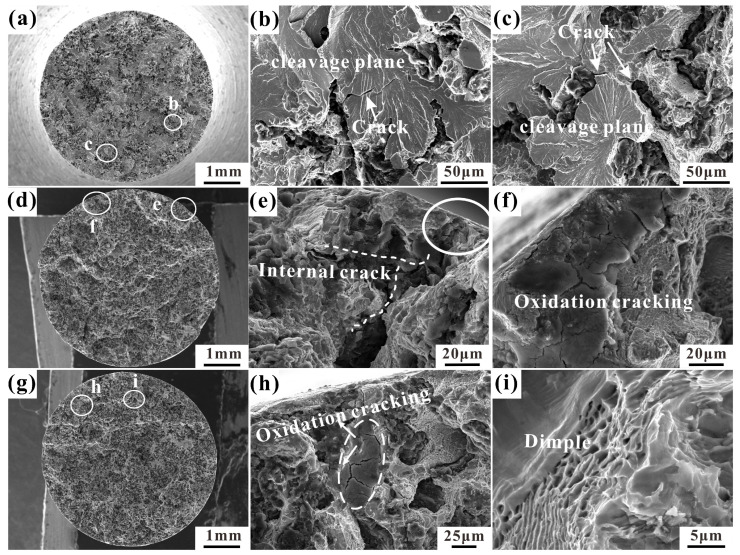
The tensile fracture morphologies of RuT450 at 25 °C, 400 °C, and 500 °C: (**a**) the whole fracture morphology at 25 °C; (**b**,**c**) are magnified images of b and c in (**a**), respectively; (**d**) the whole fracture morphology at 400 °C; (**e**,**f**) are magnified images of e and f in (**d**), respectively; (**g**) the whole fracture morphology at 500 °C; (**h**,**i**) are magnified images of h and i in (**g**), respectively.

In order to further explore the tensile properties of RuT450 at different temperatures, the tensile normal fractures at 25 °C, 400 °C, and 500 °C were selected for observation. The typical fracture at 25 °C is shown in Figure 5. The obvious river pattern cleavage surface (Figure 5a) can be observed macroscopically, accompanied by small shallow dimples and tearing edges (Figure 5b,c). The debonding between graphite units at the compacted graphite causes microcrack initiation (Figure 5c). As the loading stress continues to increase, the crack expands to the graphite/matrix interface, causing the matrix to deform. There is a slight debonding between the graphite and the matrix. When the temperature rises to 400 °C, the typical normal fracture is shown in Figure 5d. A layer of fragments covered by the oxide film appears on the fracture surface (Figure 5f), and partial oxidation cracking appears at the edge. After cracking from the edge of the graphite, the crack propagates in different directions along the inside of the graphite. Compared with the fracture at 25 °C, the cracking inside the graphite is more serious. Additionally, small amounts of tearing ridges and small dimples are present around the graphite (Figure 5e).

When the temperature is further increased to 500 °C, the typical fracture morphology is shown in Figure 5g. At this temperature, the oxidation cracking of the matrix becomes more pronounced (Figure 5h), leading to the appearance of a large number of secondary cracks. Although the cleavage plane of the entire fracture decreases, numerous deep dimples emerge (Figure 5i), the gradient of the tearing surface intensifies, and the tearing edge becomes more prominent. In summary, the tensile fracture cracks of RuT450 at different temperatures all initiate at the edge or inside of graphite and propagate through graphite clusters. At 25 °C, the cleavage plane is predominant; at higher temperatures, the number and depth of dimples increase, and the fracture of the matrix and graphite is more serious.

### 3.3. LCF Properties

**Figure 6:**
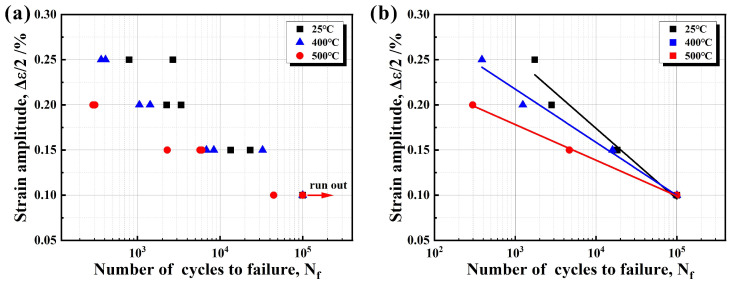
The strain amplitude–fatigue life relation of RuT450 at 25 °C, 400 °C, and 500 °C: (**a**) tested data; (**b**) mean value.

The LCF lives of RuT450 at 25 °C, 400 °C, and 500 °C are presented in Table 2 and Figure 6a. Due to the relative dispersion of the experimental data, the average value of the logarithm fatigue life was utilized (Figure 6b). At the same temperature, the fatigue life decreases as the strain amplitude increases. Under the same strain amplitude, higher experimental temperatures lead to a shorter fatigue life, with a significant decrease at 500 °C. By fitting the data, it is evident that the slope of the fatigue life curve at high temperatures is significantly lower than that at 25 °C. This indicates a significant impact of temperature variation on the LCF properties. In practical applications, temperature factors need to be thoroughly considered to ensure the reliability and safety of materials under complex conditions. This will be further elaborated below.

### 3.4. Fatigue Fracture Morphology

**Figure 7:**
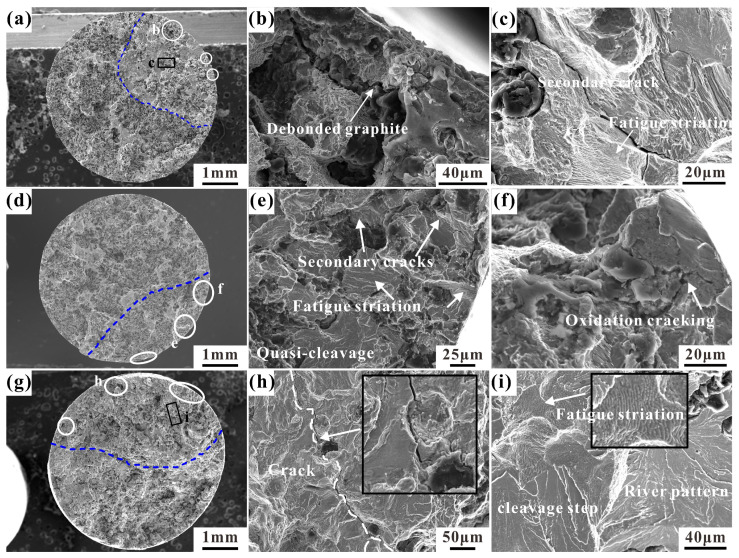
Typical fatigue fracture morphology of RuT450 at 25 °C ($\varepsilon_{t}$= 0.2%, N_f_ = 3382), 400 °C ($\varepsilon_{t}$ = 0.15%, N_f_ = 32,139), and 500 °C ($\varepsilon_{t}$ = 0.15%, N_f_ = 5707): (**a**) the whole fracture profile is at 25 °C, where the white circle is the fatigue source and the blue dotted line is the fatigue propagation zone; (**b**,**c**) are magnified images of b and c in (**a**), respectively; (**d**) the whole fracture profile is at 400 °C, where the white circle is the fatigue source and the blue dotted line is the fatigue propagation zone; (**e**,**f**) are magnified images of e and f in (**d**), respectively; (**g**) the whole fracture profile at 500 °C, where the white circle is the fatigue source and the blue dotted line is the fatigue propagation zone; (**h**,**i**) are magnified images of h and i in (**g**), respectively.

The fracture morphology of the fatigue sample is fundamental for fatigue fracture analysis. Typical LCF samples at three temperatures were selected for observation. Figure 7a–c show the fracture morphology at 25 °C. Multiple fatigue sources (white circles in Figure 7a) are visible at the edge of the fracture, indicating multi-source fatigue cracking. The fatigue expansion zone is marked by the blue dotted line. The graphite at the edge peels off the matrix to form a crack source, and the crack propagates along the tip of the graphite, as shown in Figure 7b. Secondary cracks extend from the graphite in the fatigue propagation zone, and fatigue striations can be found in the matrix, as shown in Figure 7c. The fracture form of the matrix is mainly quasi-cleavage fracture. Figure 7d–f show the LCF fracture surface at 400 °C. Fatigue striations shown in Figure 7e appear in the crack propagation zone, and the quasi-leavage plane of the river pattern is found in a local part of the matrix, indicating that the fatigue propagation process is a mixed form. The high-temperature oxidation phenomenon in Figure 7f appears at the edge of the fracture, and the cracks at the fatigue source are covered by the oxide film. Figure 7g shows the LCF fracture morphology at 500 °C. In Figure 7h, it can be observed that the longer secondary crack propagates with graphite as the medium (see the inset). The cleavage planes and cleavage steps of river patterns and the fatigue striations can be observed in Figure 7i. It shows that in the LCF fracture of CGI, the fracture is a mixed fracture mode composed of the stable propagation of the fatigue crack and local quasi-cleavage cracking.

## 4. Discussion

### 4.1. Cyclic Stress Response Curves

**Figure 8:**
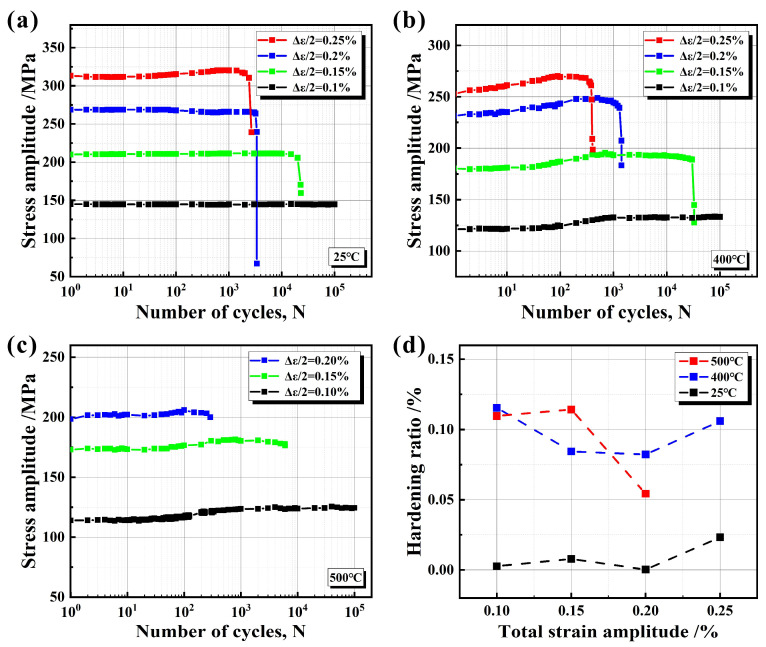
The cyclic stress response curves of RuT450 at (**a**) 25 °C, (**b**) 400 °C, and (**c**) 500 °C and (**d**) the relationship between cyclic hardening rate and temperature and strain amplitude.

Figure 8a–c show the cyclic stress response curves of tension−compression LCF at 25 °C, 400 °C, and 500 °C. At three temperatures, with the increase in strain amplitude, the initial strain amplitude increases accordingly; on the other hand, the fatigue life shows a decreasing trend. The cyclic stress response curves at 25 °C are shown in Figure 8a. It can be found that under the condition of low strain amplitude, the cyclic stress shows good stability, and no obvious change can be seen in the cyclic stress response curve. However, when the strain amplitude increases to 0.25%, the cyclic hardening phenomenon begins to appear. When the temperature rises to 400 °C, the cyclic stress response curves (Figure 8b) exhibit different characteristics. At low strain amplitudes (0.1%, 0.15%), the initial cycle remains stable, but close to 100 cycles, the cyclic hardening phenomenon begins to appear. Under the condition of high strain (0.2%, 0.25%), cyclic hardening is particularly obvious from the initial stage, with the highest cyclic hardening rate observed at a strain amplitude of 0.25%. Further observation of the cyclic stress response curves at 500 °C (Figure 8c) reveals that due to the extremely short LCF life of the material under the high-strain condition of 0.25% during the experiment, the cyclic response curve under this strain does not provide reference value for the analysis of experimental data, so the experimental data are not listed.

In order to describe the cyclic phenomenon in the process of LCF more intuitively, the cyclic hardening rate was used (Figure 8d). It can effectively reflect the change in stress during cyclic loading, and then reveal the extent and pattern of cyclic hardening. Its expression is:

(2) $$HR=(∆\sigma_{max}-∆\sigma_{first})/∆\sigma_{first}$$

Here, $∆\sigma_{\max}$ and $∆\sigma_{first}$ represent the maximum stress amplitude and the initial stress amplitude in the cyclic stress response curve, respectively.

For the cyclic hardening rate at 25 °C, when the strain increases to 0.25%, the hardening rate increases significantly. This is consistent with the phenomenon shown in Figure 8a. Compared with 25 °C, the degrees of cyclic hardening at 400 °C and 500 °C are greater, as clearly reflected in Figure 8d. In the LCF experiment, vermicular graphite particles peel off to form microcracks, and the stress concentration at the crack tip causes the strengthening of the adjacent matrix. In addition, the precipitation of carbides at high temperatures and the large plastic zone area under high strain amplitude lead to cyclic hardening in the process of macroscopic cyclic deformation. The cyclic hardening amplitude is related to the matrix content and strengthening ability involved in the deformation.

### 4.2. Hysteresis Loops

**Figure 9:**
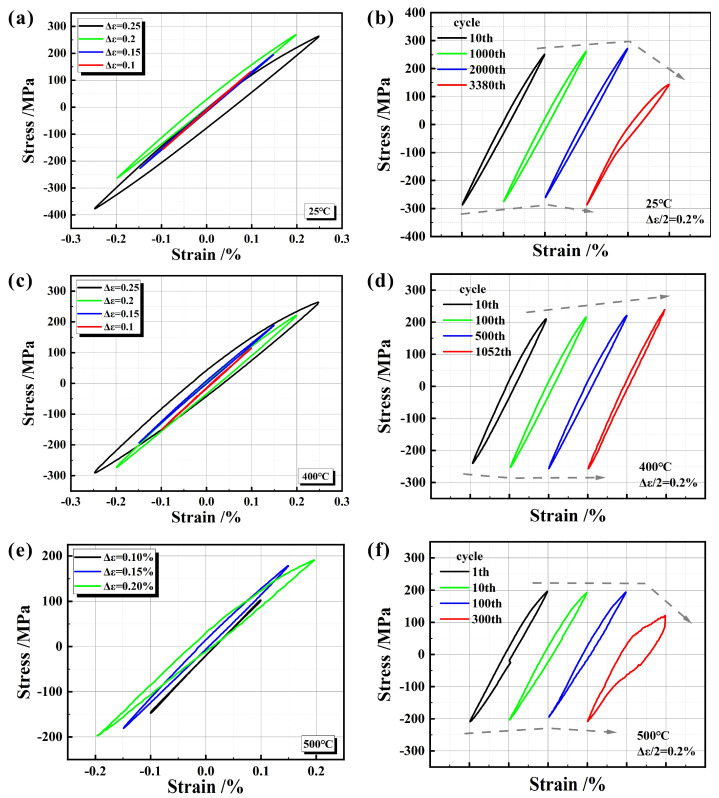
Half−life hysteresis loops: (**a**) 25 °C, (**c**) 400 °C, and (**e**) 500 °C; hysteresis loops of different cycles at strain amplitude of 0.2 % (**b**) 25 °C, (**d**) 400 °C, and (**f**) 500 °C.

The half-life hysteresis loops of the three temperatures at different strain amplitudes are shown in Figure 9a,c,e. As the total strain amplitude increases, the stress amplitude also increases, and the area of hysteresis loops (hysteresis energy) at the three temperatures increases. During the high-strain cyclic loading process at 25 °C, there is a noticeable tension−compression asymmetry, where compressive stress exceeds tensile stress. This phenomenon is primarily attributed to the limited adhesion between the brittle graphite and the matrix, leading to its tearing or debonding from the matrix during the tensile stress process. Under the same total strain amplitude at different temperatures, the stress amplitude decreases as the temperature rises.

Figure 9b,d,f describe the typical hysteresis loops of the three temperatures at different cycles with a total strain amplitude of 0.2%. At 25 °C, as the number of cycles increases, the hysteresis loops move slightly upward (the tensile stress increases and the compressive stress decreases), but the final stress amplitude decreases, and a deflection phenomenon appears in the compression part of the hysteresis loop. At 400 °C, with an increase in the cycle number, the hysteresis energy shows a decreasing trend, the overall compressive stress remains stable, while the tensile stress increases. At 500 °C, as the number of cycles increases, the hysteresis loops initially remain stable, but the final stress amplitude decreases, and the hysteresis phenomenon becomes more serious. This is attributed to the softening of the matrix at high temperatures, which weakens the self−recovery ability of the material.

### 4.3. Crack Growth and Fracture Profiles

**Figure 10:**
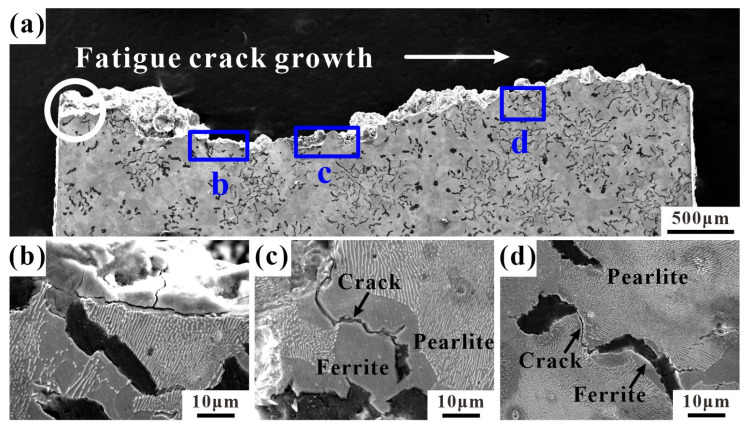
The longitudinal section of a typical fracture of RuT450 at 25 °C: (**a**) the whole fracture profile of the failure sample ($\varepsilon_{t}$ = 0.25%, N_f_ = 2682), and the white circle is the source of fatigue; (**b**–**d**) the magnified images of b, c, and d in (**a**), respectively.

In order to further analyze the LCF fracture mechanism of RuT450, the longitudinal section microstructures of LCF samples at three temperatures were observed. Figure 10 shows the longitudinal section of the LCF fracture of the sample at 25 °C. The fatigue source of the sample is in the graphite cluster indicated by the white circle at the edge, which extends along the arrow direction in Figure 10a. The enlarged observations at (b) and (c) in Figure 10a show that the main crack propagates along the graphite in the ferrite, and the matrix at the edge of the fracture is partially broken. Figure 10d reveals a small number of fine secondary cracks inside the pearlite, primarily extending through the ferrite.

**Figure 11:**
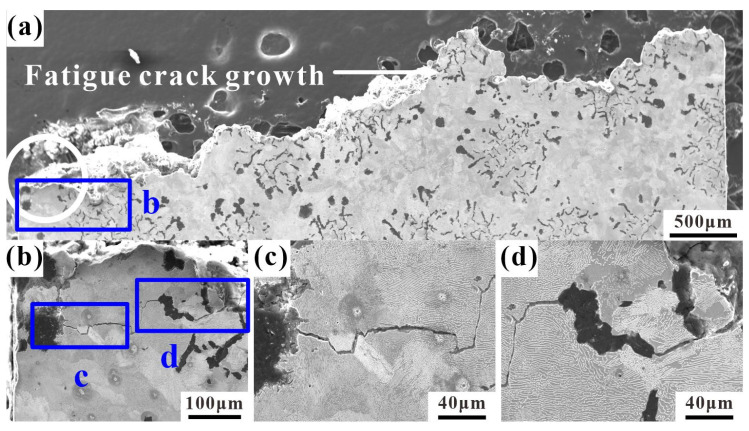
The longitudinal section of a typical fracture of RuT450 at 400 °C: (**a**) the whole fracture profile of the failure sample ($\varepsilon_{t}$ = 0.2%, N_f_ = 1421) and the white circle is the source of fatigue; (**b**) magnified image of b in (**a**); (**c**,**d**) magnified images of c and d in (**b**), respectively.

**Figure 12:**
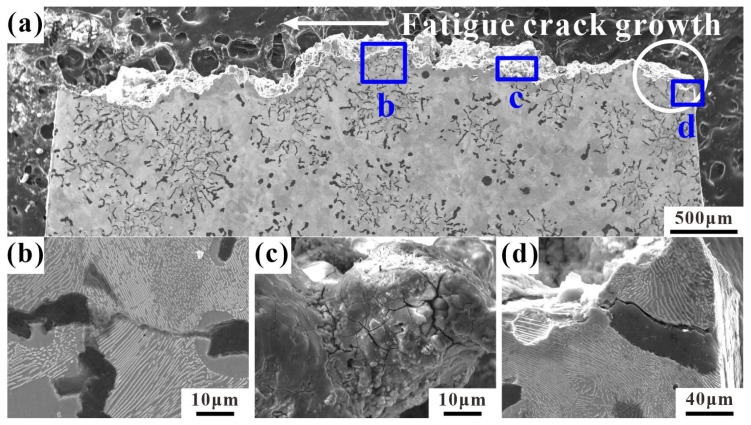
The longitudinal section of a typical fracture of RuT450 at 500 °C: (**a**) the whole fracture profile of the failure sample ($\varepsilon_{t}$ = 0.15%, N_f_ = 6030) and the white circle is the source of fatigue; (**b**), (**c**,**d**) magnified images of b, c, and d in (**a**), respectively.

Figure 11 shows the longitudinal section of the LCF fracture of the sample at 400 °C. The fatigue source is in the graphite cluster within the white circle at the edge of the fracture and extends along the direction of the white arrow, as shown in Figure 11a. The enlarged observation of (b) in Figure 11a shows obvious longer cracks near the fatigue source, extending from the inside of the graphite to the matrix. The enlarged observations in (c) and (d) in Figure 11b show that the crack propagation at this stage differs from that at 25 °C. When the crack passes through the compacted graphite, it propagates along the interface between the graphite and the surrounding matrix, and the crack also propagates inside the pearlite. Oxidation occurs on the inner surface of the crack and at the interface between the compacted graphite and the ferrite matrix, mainly invading the interior matrix through the gap between the compacted graphite and the matrix (Figure 11c), and the crack is not completely covered by the oxide. Figure 12 shows the longitudinal section of the LCF fracture of the sample at 500 °C. From the macroscopic view of the longitudinal section in Figure 12a, the fatigue source is also in the graphite cluster in the white circle at the edge, which expands along the direction of the white arrow. In Figure 12d, there is obvious cracking between graphite and the matrix. On one hand, due to the cyclic load, the adhesion between the graphite and the matrix is poor, causing it to tear under tensile stress. On the other hand, at high temperatures during oxidation, it is easier for the gap between the graphite and the matrix to be invaded, which leads to obvious debonding of graphite. By observing the enlarged image in (c) in Figure 12a, it is evident that the oxidation cracking at the edge of the fracture is obviously aggravated at a high temperature of 500 °C, and the oxide layer has completely covered the surface. At this time, the dependence of the crack propagation path on ferrite obviously reduces, and a large number of secondary cracks can be found in the adjacent pearlite around the graphite.

### 4.4. Damage Mechanism

**Figure 13:**
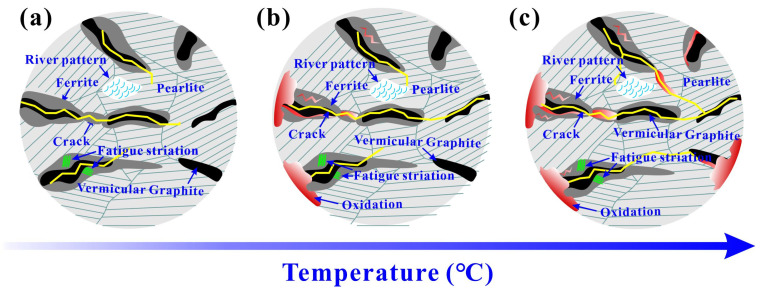
The illustration of LCF deformation behaviors of RuT450 at (**a**) 25 °C, (**b**) 400 °C, and (**c**) 500 °C.

The LCF damage mechanism of RuT450 at different temperatures is illustrated in Figure 13. At 25 °C (Figure 13a), pearlite and ferrite play different roles in the fatigue fracture process. The inhibition of Fe_3_C on dislocation slip in pearlite leads to the difficulty of crack initiation. Compared with the graphite/pearlite interface, the debonding of the graphite/ferrite interface is more likely to cause fatigue crack initiation. Under cyclic loading, cracks continue to expand in ferrite. It is worth noting that during the LCF crack propagation in CGI, not only were obvious fatigue striations found in the ferrite region around graphite, but also, the quasi-cleavage surfaces of river-like patterns were found on the pearlite matrix, indicating that the LCF was a mixed fracture mode composed of stable fatigue crack propagation and local quasi-cleavage cracking. In addition, the propagation direction of the crack is transmitted from the tip of one graphite to the tip of another graphite. Secondary cracks along the direction perpendicular to the main crack propagation direction also emerge in the form of graphite tip penetration. When the temperature rises to 400 °C in Figure 13b, the mechanical properties of pearlite decrease at high temperature, the bearing capacity of the matrix decreases, and the dependence on ferrite decreases during crack propagation. Crack propagation can also be observed in pearlite. Partial oxidation occurs at the edge and inside of the sample. The formation of an oxide film on the crack surface and tip causes the crack tip to become brittle, and it is easy to crack under large strain amplitude. It is well known that vacancies diffuse to grain boundaries at high temperatures, resulting in grain boundary sliding, which reduces crack propagation resistance and leads to a decrease in cyclic stress amplitude. This phenomenon is more pronounced in large strain amplitudes. At 500 °C (Figure 13c), crack propagation will occur along the fracture surface with more graphite and interface due to the sliding of ferrite grain boundaries and pearlite colony interfaces. Therefore, the pearlite content in the crack propagation path in Figure 13c is significantly higher than that at 25 °C and 400 °C. The increase in oxidation degree further reduces the bearing capacity of the matrix, and these structural changes lead to the decrease in LCF life.

### 4.5. Prediction of LCF Life

**Figure 14:**
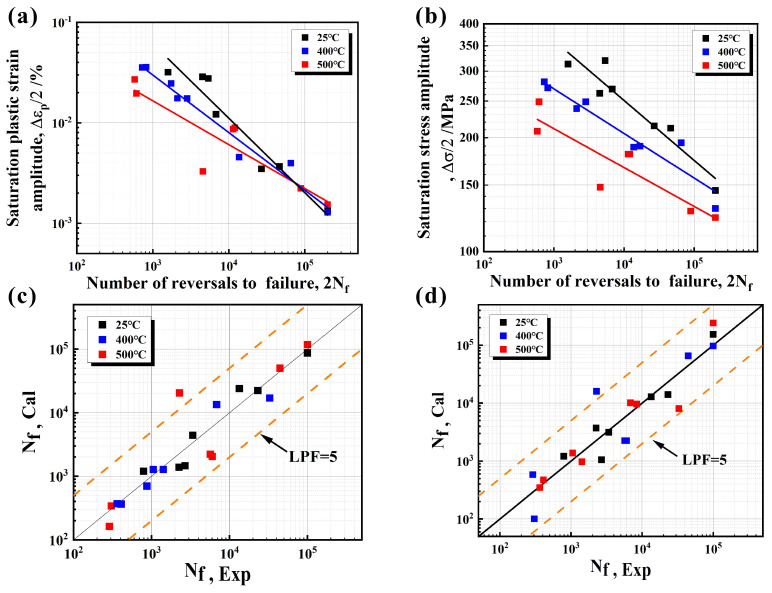
(**a**) Coffin−Manson curves and (**c**) prediction results; (**b**) Basquin curves and (**d**) prediction results.

Before fatigue fracture, CGI does not exhibit obvious plastic deformation, which is difficult to detect and prevent. Therefore, the fatigue fracture of materials and components will cause huge economic losses and pose a great threat to personal safety. Thus, it is of great significance to study the fatigue problem, especially the prediction of fatigue life. The LCF damage process of CGI is relatively complex. And so, the established life prediction model not only accurately predicts the trend of LCF life, but also comprehensively considers the difference in damage mechanisms at different temperatures, enabling a more reasonable evaluation of the LCF performance of CGI. The traditional Coffin−Manson model is only based on plastic strain, and the Basquin model is mainly based on cyclic stress [25,26]. Both were used to predict the LCF life of RuT450. The results are shown in Figure 14, and neither of them can accurately reflect the overall fatigue performance, including stress and strain. The life prediction factor (LPF) [35] is usually used to evaluate the ability of a life prediction method, namely:

(3) $$\mathrm{LPF}=\max\left\{\frac{N_{\mathrm{cal}}}{N_{\exp}},\;\frac{N_{\exp}}{N_{\mathrm{cal}}}\right\}$$

N_cal_ and N_exp_ are the fatigue life obtained through calculation and experimentation, respectively. The prediction results show that the LPF of the Coffin–Manson and Basquin models are both greater than 5, and the prediction error is poor, making them unsuitable for the prediction of LCF life of RuT450.

In recent years, the energy method has been widely used to analyze the LCF properties of materials from an energy point of view [30,35,36]. The energy method holds that the material absorbs part of the energy acting on it and converts it into internal fatigue damage during each loading process. The greater the total energy applied to the material, the greater the energy of the material transformed into fatigue damage. The fatigue life prediction model based on the energy method has been successfully applied in predicting the fatigue life of various materials due to its concise parameters, clear physical significance, and accurate predictions [31]. The energy cumulative damage model is as follows:

(4) $$D_{i}={\left(\frac{W_{i}}{W_{0}}\right)}^{\beta }$$

(5) $$D=\sum_{i=1}^{N_{f}}D_{i}=\sum_{i=1}^{N_{f}}{\left(\frac{W_{i}}{W_{0}}\right)}^{\beta }=1$$

Here, *D*_i_ is the damage parameter of the i-th cyclic loading; *W*_i_ is the hysteresis energy of the i-th loading, MJ/m^3^; *W*_0_ is the inherent fatigue toughness of the material; *β* is the damage transition index; *D* is the sum of the fatigue damage of the material, MJ/m^3^. When its value is equal to 1, the material breaks. When *D* = 1, the Equation (4) can be transformed into:

(6) Ws=W0·Nf−1β

Here, *W*_s_ is the medium-life hysteresis energy, MJ/m^3^.

**Figure 15:**
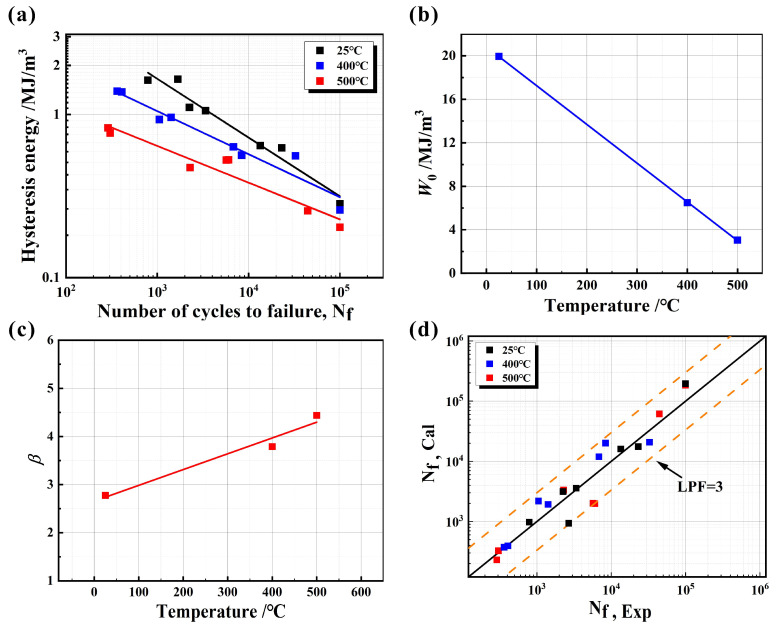
LCF life prediction by energy model: (**a**) the relationship between median hysteretic energy and fatigue life at different temperatures; linear fitting of temperature with (**b**) *W*_0_ and (**c**) *β*; (**d**) comparison of experimental and predicted fatigue life.

The relationship between half-life hysteresis energy and fatigue life in double logarithmic coordinates is shown in Figure 15a. The fatigue life of CGI increases linearly at different temperatures, while the median hysteresis energy decreases, which is consistent with the change in life with strain amplitude. The values of *W*_0_ and *β* can be obtained separately from the fitting results, and their relationship with temperature is shown in Figure 15b,c. Wang et al. [37] and Zou et al. [35] also found these relations. We fit them linearly as follows:

(7) $$W_{0}=\mathrm{aT}+b$$

(8) β=mT+n

After fitting, the values of constants a, b, m, and n are a = −0.0275, b = 16.3483, m = 0.0036, and n = 2.9432, respectively. The energy cumulative damage model at different temperatures is obtained as follows:

(9) Ws=aT+b·Nf−1(mT+n)

The predicted results based on the calculation results of Equation (9) and the experimental results of Table 1 are shown in Figure 15d. It can be concluded that the model demonstrates good adaptability to the experimental results within 3 times the predicted life (LPF = 3). It is more accurate than the Coffin−Manson and Basquin models mentioned above (Figure 14c,d).

The prediction results (Figure 15d) indicate that the prediction accuracy at 25 °C is significantly better than that at high temperatures. On the one hand, because there are many instabilities in the casting process, the graphite morphologies in the CGI cut from different components are diverse, and the fatigue source (Figure 7b,f) will preferentially initiate and expand at the graphite tip. The uniformity of its size and distribution has a great influence on the fatigue life. On the other hand, at high temperatures, the strength of CGI decreases, which leads to the increase in plastic deformation, which further aggravates the uniformity of its microstructure, increases the dispersion of fatigue life, and complicates the prediction of fatigue life at these temperatures.

## 5. Conclusions

In this study, the tensile and LCF performances of RuT450 were investigated at different temperatures. Different cyclic stress responses and fatigue fractures were observed and analyzed at 25 °C and high temperatures. The LCF damage mechanism of CGI at different temperatures and the corresponding fatigue life prediction model were discussed. Based on the experimental results and analysis above, the following conclusions can be obtained:

1. With the increase in experimental temperature, the yield ratio of RuT450 increases, and dimples appear in the tensile fracture at high temperatures. The decreasing trend of LCF life at 500 °C was significantly higher than those at 25 °C and 400 °C.
2. At 25 °C, the fatigue crack initiates from the graphite tip and propagates along the inside or edge of the ferrite. At 400 °C, a layer of oxide film appears at the edge of the sample and around the crack, and the crack propagates along the ferrite and pearlite. At 500 °C, the oxidation cracking of graphite and matrix is obvious, and the dependence of crack propagation on ferrite greatly reduces.
3. The LCF life prediction model, based on the linear relationships of the energy method parameters, can more effectively predict the LCF life of CGI compared with the other two models.

## Figures and Tables

**Table 1:** The chemical composition of RuT450 (wt.%).

C	S	Si	Mn	Cu	Cr	Sn	Mo	Re	Mg
3.81	0.01	2.20	0.32	0.80	0.05	0.09	0.001	0.02	0.017

**Table 2:** Results of fatigue lives in LCF tests of RuT450 at 25 °C, 400 °C, and 500 °C.

Test Temperature/°C	Total Strain Amplitude ∆ε_t_/2/%								
	0.1		0.15			0.2		0.25	
25	10^5^	-	13,427	23,136	-	3382	2254	2682	791
400	10^5^	-	32,739	6833	8387	1053	1421	363	409
500	10^5^	44,508	5707	6030	2291	289	305	-	-

## Data Availability

Data are contained within the article.
